# SH1-dependent maize seed development and starch synthesis via modulating carbohydrate flow and osmotic potential balance

**DOI:** 10.1186/s12870-020-02478-1

**Published:** 2020-06-08

**Authors:** Ke Zhang, Li Guo, Wen Cheng, Baiyu Liu, Wendi Li, Fei Wang, Changzheng Xu, Xiangyu Zhao, Zhaohua Ding, Kewei Zhang, Kunpeng Li

**Affiliations:** 1 The Key Laboratory of Plant Development and Environment Adaptation Biology, Ministry of Education, School of Life Science, Shandong University, Qingdao, 266237 China; 2 Maize Institute of Shandong Academy of Agricultural Sciences, Jinan, Shandong China; 3 National Key Laboratory of Plant Molecular Genetics, CAS Center for Excellence in Molecular Plant Sciences, Institute of Plant Physiology and Ecology, Shanghai Institutes for Biological Sciences, Chinese Academy of Sciences, Shanghai, 200032 China; 4 School of Life Sciences, Southwest University, Chongqing, 400715 China; 5 State Key Laboratory of Crop Biology, College of Life Sciences, Shandong Agricultural University, Taian, 271018 Shandong China

**Keywords:** Glucose-1-phosphate, Seed development, *SH1*, Soluble sugars, Starch synthesis, *Zea mays*

## Abstract

**Background:**

As the main form of photoassimilates transported from vegetative tissues to the reproductive organs, sucrose and its degradation products are crucial for cell fate determination and development of maize kernels. Despite the relevance of sucrose synthase SH1 (shrunken 1)-mediated release of hexoses for kernel development, the underlying physiological and molecular mechanisms are not yet well understood in maize (*Zea mays*).

**Results:**

Here, we identified a new allelic mutant of *SH1* generated by EMS mutagenesis, designated as sh1*. The mutation of *SH1* caused more than 90% loss of sucrose synthase activity in sh1* endosperm, which resulted in a significant reduction in starch contents while a dramatic increase in soluble sugars. As a result, an extremely high osmolality in endosperm cells of sh1* was generated, which caused kernel swelling and affected the seed development. Quantitative measurement of phosphorylated sugars showed that Glc-1-P in endosperm of sh1* (17 μg g^− 1^ FW) was only 5.2% of that of wild-type (326 μg g^− 1^ FW). As a direct source of starch synthesis, the decrease of Glc-1-P may cause a significant reduction in carbohydrates that flow to starch synthesis, ultimately contributing to the defects in starch granule development and reduction of starch content.

**Conclusions:**

Our results demonstrated that SH1-mediated sucrose degradation is critical for maize kernel development and starch synthesis by regulating the flow of carbohydrates and maintaining the balance of osmotic potential.

## Background

Maize kernel development is a dynamic and sophisticated developmental process orchestrated by complicated regulatory networks involving a large number of genes. Owing to molecular and genetics studies, many genes that are essential for kernel development have been identified, including those involved in basal endosperm transfer layer (BETL) differentiation [1–3], sugar transportation [4–7], sucrose metabolism and starch synthesis [8–12].

As a major organ for nutrition storage, kernels assimilate a large amount of carbohydrates that are produced in photosynthetic cells. Apart from energy supply, most of those carbohydrates are converted into starch, which accounts for 70–75% of seed weight [13, 14]. Sucrose is the main form of photoassimilates that is transported from photosynthetic cells to kernels [4–6, 15]. The families of sucrose transporters (SUTs) and SWEET proteins, have been shown to participate in sucrose transportation [7]. SUTs are the major sucrose transporters [7], while SWEETs constitute a new hexose and sucrose transporters family. In maize, *ZmSWEET4c* is mainly expressed in the BETL, the entry point of sucrose into seed, and is thereby critical for BETL formation and seed filling [1]. During seed development, sucrose is not only the raw material for cell wall formation, starch synthesis and glycolysis, but also an important signaling regulator of hormonal signaling and cell fate determination by affecting the expressions of related genes [12, 16]. Several studies have shown that the expression of multiple key genes, such as *ZmMRP-1*, *ZmYUC1*, cell wall invertase *MINIATURE1* (*Mn1*) and *ZmSWEET4c*, is modulated by the content of sugars including sucrose and its degradation products during early kernel development, and function in regulation of BETL cells differentiation and kernel development [1, 2].

Sucrose catabolism mainly depends on two enzyme families: invertase (INV) and sucrose synthase (SUS) [16–19]. INVs catalyze the irreversible decomposition of sucrose into glucose and fructose, which are the substrates of glucose-6-phosphate (Glc-6-P) and fructose-6-phosphate (Fru-6-P), the important precursors for multiple downstream pathways [20]. Six invertases have been annotated in maize genome so far [21]. Among them, INCW2 encoded by *Mn1* has been identified as a BETL-specific protein [15, 22–24]. The loss-of-function of *Mn1* resulted in higher sucrose content and lower hexose to sucrose ratio in endosperm cells, and 70% loss of seeds weight [22, 24]. Gene expression analysis indicated several key genes involved in starch synthesis (*Sh2* and *Bt2*) and encoding sucrose synthase (*SH1*, *SUS1* and *SUS2*) were significantly down-regulated in the *mn1* mutant [24]. These data indicated that abnormal sucrose metabolism caused by *Mn1* mutation led to differential expression of a large number of genes related to carbon metabolism, which in turn affected maize seed development and yield.

In contrast with INVs, functional characterization of sucrose synthase is relatively lacking during seed development in maize. SUSs can reversibly transform sucrose into fructose and uridine-diphosphoglucose (UDP-Glc). UDP-Glc acts as a substrate for cellulose synthesis, the concentration of which can affect cell wall formation. In addition, UDP-Glc can be converted into Glc-1-P, a carbon source for starch synthesis, which is catalyzed by UDP-glucose pyrophosphorylase. Therefore, SUSs are considered to play important roles in cell wall formation and starch synthesis [16–18]. In maize genome, 20 genes were predicted to encode sucrose synthase, three of which including *SUS1*, *SUS2*, and *SH1* have been functionally identified [25]. *SUS2* encoding a ‘housekeeping’ SUS isozyme is localized in the cytoplasm and cleaves sucrose for cytoplasmic metabolism [26]. *SH1* and *SUS1* encode two biochemically similar isozymes [27]. Both of them, unlike SUS2, were proven to be associated with membranes, implying their distinct functions from SUS2 [9, 26]. Previous studies have shown that the loss-of-function of *SH1* resulted in a significant reduction of sucrose synthase activity and a decreased starch accumulation, thereby leading to shrunken kernels [28]. The starch contents in kernels of *sh1 SUS1* and *sh1 sus1–1* genotypes are 78 and 53% of *SH1 SUS1*, respectively. Chourey et al. (1998) deemed that *SH1* and *SUS1* predominately functioned in cellulose biosynthesis and starch biosynthesis, respectively [8, 27]. The functional loss of *SH1* caused the restriction of UDP-Glc into cellulose biosynthesis during cell elongation [8]. A recent study confirmed that *SH1* also played an important role in starch synthesis, and *SH1* null mutation resulted in a significant increase in the ratio of amylose to amylopectin in the endosperm [11]. These above studies provided a preliminary understanding of the function of *SH1* in maize seed development. However, the roles of sucrose degradation pathway catalyzed by *SH1* in maintenance of carbon metabolism balance and regulation of gene expression during seed development are not yet fully understood.

In the present study, we revealed the roles of *SH1* in appropriate carbon partitioning, maintaining the balance of osmotic potential, regulating the starch synthesis and seed development via characterization of sh1* mutant. Null mutation of *SH1* led to less carbohydrates flowing to starch synthesis pathway. A large number of carbohydrates exist in the form of soluble sugars. The carbon metabolic disorder induced by *SH1* mutation leads to the kernel development arrest and shrunken phenotype in the sh1* mutant.

## Results

### sh1* produces shrunken kernels with reduced starch contents and dysplastic starch granules

**Fig. 1:**
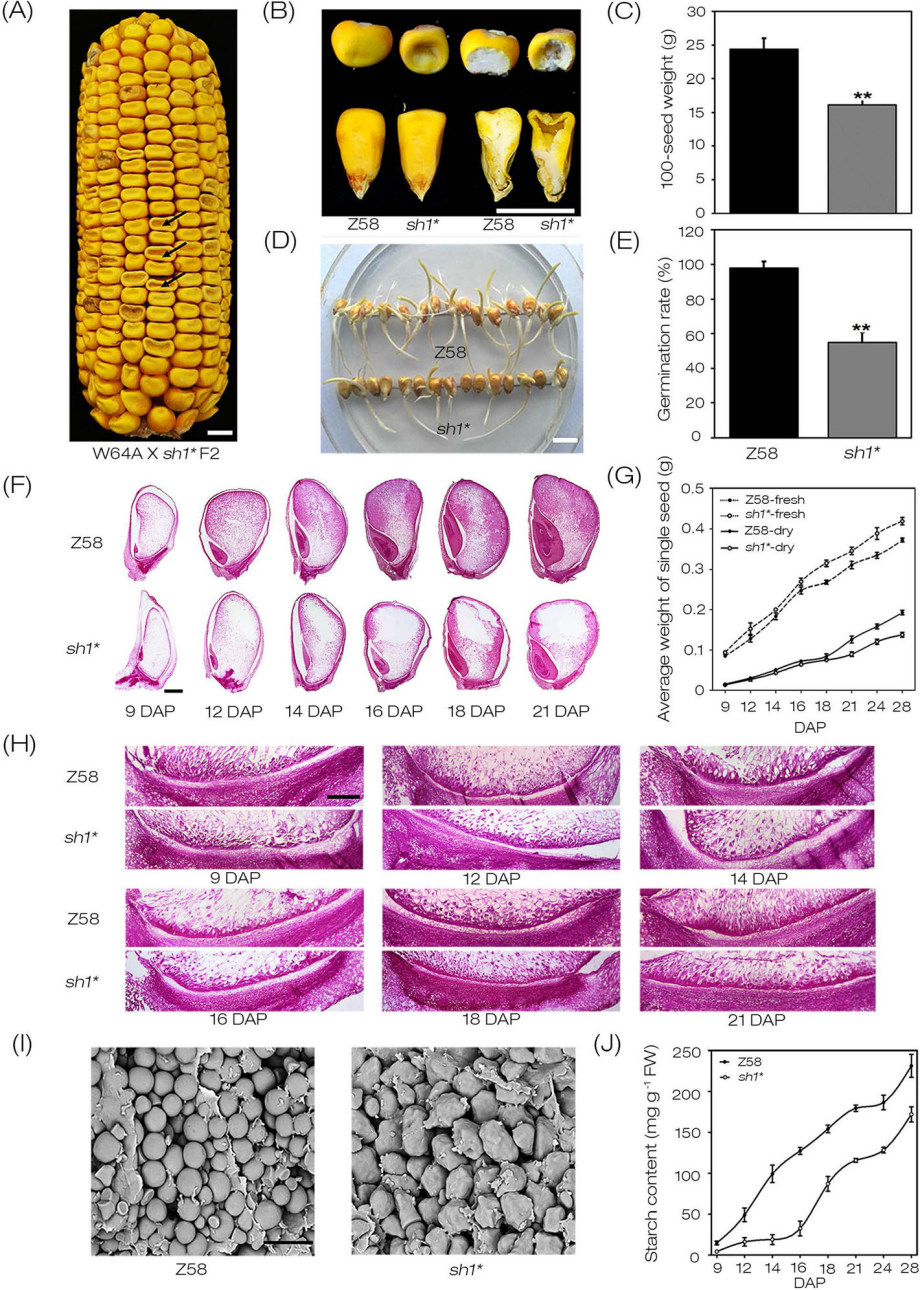
Phenotype characterization of sh1*. **a** Mature F2 ear of W64A × sh1*, the arrow identified the sh1* kernels. Bar = 0.5 cm. **b** Observation of complete (left) and dissected (right) mature kernels of WT and sh1*. Bar = 1 cm. **c** Comparison of 100-mature kernels weight of randomly selected Z58 and sh1* kernels. Values were means with SD. (**, *P* < 0.01, student’s t test). **d** Germination experiment of mature kernels of Z58 and sh1*. Bar = 1 cm. **e** Comparison of germination rate of Z58 and sh1* kernels. Values were means with SD. (**, *P* < 0.01, student’s t test). **f** Paraffin sectioning of Z58 and sh1* kernels during 9 to 21 DAP. Bar =1 mm. **g** Comparison of fresh- and dry-weight of Z58 and sh1* kernels during 9 to 28 DAP. The data showed were the average of three independent experiments and error bars represent SD. **h** Comparison of basal endosperm transfer layer (BETL) in Z58 and sh1* kernels during 9 to 21 DAP. Bar = 20 μm. **i** Microstructure of mature starch granules in Z58 and sh1* kernels. Bar = 500 μm. **j** Comparison of starch content from Z58 and sh1* developing kernels. Three times of starch content measurement were done, and the values were means with SD

The sh1* mutant was obtained by ethyl methanesulfonate (EMS) mutagenesis. It was crossed with W64A to produce an F2 genetically-isolated population that displayed a 3:1 segregation of wide-type (+/+ and sh1*/+) and shrunken (sh1*/sh1*) phenotypes (Fig. 1a). Compared with Z58, kernels of the sh1* were badly concave in the middle of the top after dehydration, and had less cytoplasmic dense inside of the kernels (Fig. 1a, b). The 100-seed weight of sh1* was about 33% less than that of Z58 (Fig. 1c). To explore the effects of the developmental defects on seed germination, germination experiments of sh1* and Z58 were performed on 1/2 MS medium. Our results showed that the germination rate of sh1* seeds was only about 54% of that in Z58 (Fig. 1d, e).

To investigate the differences in kernels development between sh1* and Z58, their kernels at 9, 12, 14, 16, 18 and 21 days after pollination (DAP) were observed. Longitudinal sections indicated that the kernel development of sh1* were significantly delayed compared with Z58. The embryos were smaller and the endosperm had dramatically less materials accumulation in sh1* at same stages than Z58 (Fig. 1f). The cell deficiency in endosperm of sh1* at 12, 14, 16, 18 and 21 DAP were observed in the centrally-located starch storage cells, except for the kernels of 9 DAP (Fig. 1f). Detection of the fresh- and dry-weight of kernels indicated that there were no significant differences between sh1* and Z58 at 9, 12, 14, 16 DAP, whereas they began to show difference after 18 DAP that became more and more notable later on during the developmental process (Fig. 1g). Although the dry-weight of sh1* kernels was significantly lower than that of Z58 at the same stage after 18 DAP, it had obviously higher fresh weights. These results indicated that, compared to Z58, sh1* kernels were able to absorb more water, but accumulate fewer dry contents. As sink organs, developing kernels obtain nutrients and storage substances from vegetative tissues, and this process relies on BETL cells. Our results indicated that at the same stages, the BETL development of sh1* kernels was relatively delayed compared to WT (Fig. 1h), which might affect the accumulation of dry matter in sh1* kernels.

Observation of sh1* and Z58 mature kernels indicated that starch granules in endosperm of Z58 kernels exhibited a regular round shape and densely distributed, whereas those of sh1* were of irregular shape and loosely arranged (Fig. 1i). The content of starch in sh1* kernels was significantly lower than that of Z58 (Fig. 1j). These results indicated that the development of kernels was weakened and the accumulation of starch was reduced in sh1*.

### Sh1* encodes the sucrose synthase SH1

**Fig. 2:**
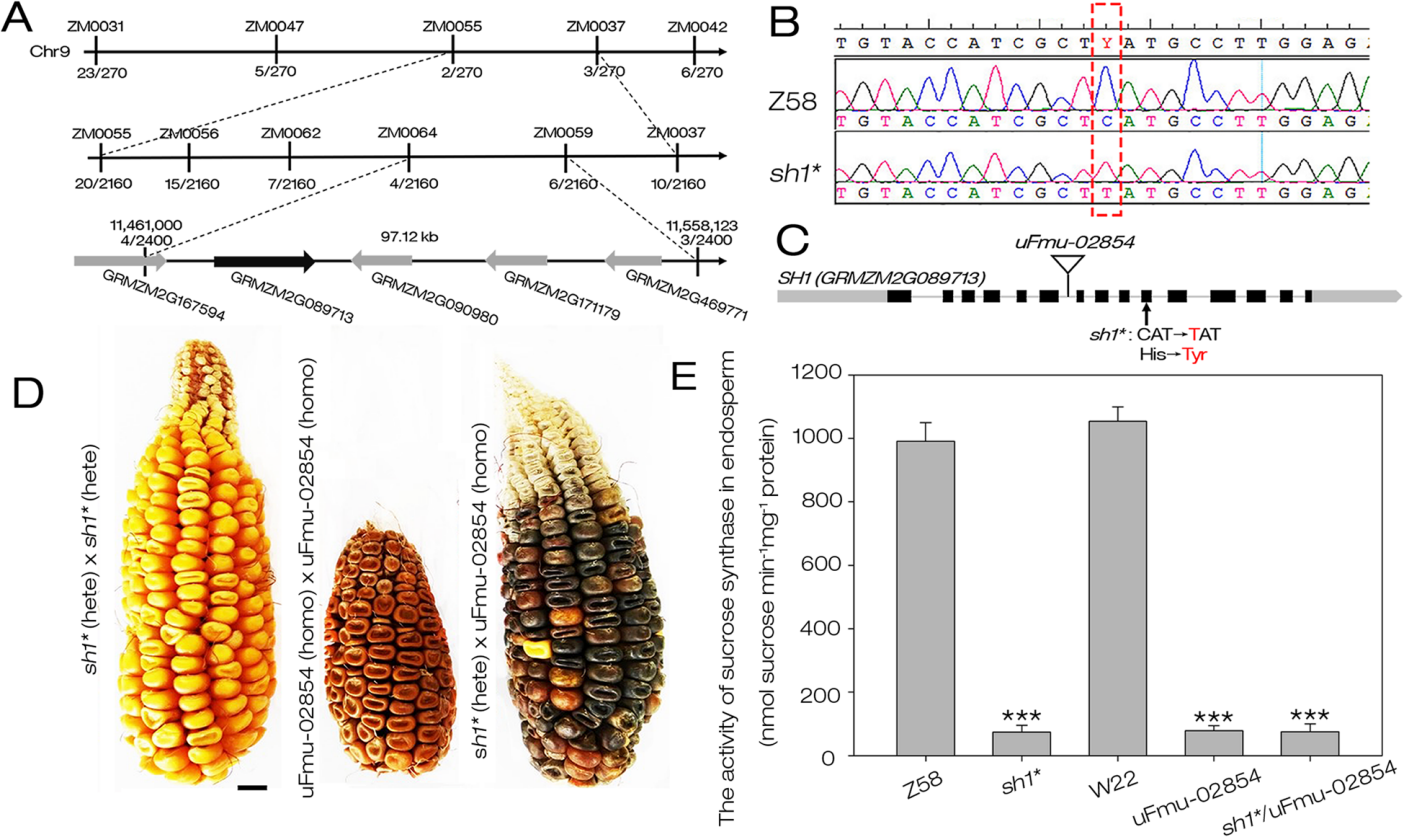
Map-based cloning and identification of sh1*. **a** The sh1* locus was mapped to a 97.12-kb interval between molecular ZM0064 and ZM0059 on chromosome 9. **b** Sequence alignment (partial) of *SH1* in Z58 and sh1*. The dotted box marked the mutation site in sh1*. **c** Gene model of *SH1.* Gray lines represent introns and black boxes represent exons, gray box and arrow represent 5’UTR and 3’UTR, respectively. Mutation site of sh1* (arrow) and insertion site of uFmu-02854 (triangle) were indicated. **d** Heterozygous sh1* and uFmu-02854 were used for allelism test of sh1*. Hete: heterozygous, homo: homologous. Bar = 0.5 cm. **e** The sucrose synthase activity of the endosperm of 16 DAP kernels of sh1*, uFmu-02854, sh1*/uFmu-02854 as well as WT controls Z58 and W22. Values were means with SD. (***, *P* < 0.001, student’s *t-*test)

Genetic fine-mapping of sh1* was performed with 2400 shrunken kernels from F2 mapping population, and the sh1* locus was localized in a region of 97.12 kb on chromosome 9 between the self-created molecular markers ZM0064 and ZM0059 (Fig. 2a). Retrieving the maizeGDB database (https://www.maizegdb.org/), we found that there are 5 predicted genes within this interval, including GRMZM2G167594, GRMZM2G089713, GRMZM2G090980, GRMZM2G171179 and GRMZM2G469771 (Fig. 2a). Nucleotide sequence analysis of Z58 and sh1* revealed a C to T substitution at 1297 bp (the “A” of the translation start codon “ATG” was designated “+ 1”) in open reading frame (ORF) of GRMZM2G089713 (Fig. 2b and Fig. S1), caused a mutation of histidine to tyrosine (Fig. 2c). The ORF sequences of GRMZM2G167594, GRMZM2G090980, GRMZM2G171179 and GRMZM2G469771 have no differences between Z58 and sh1*. Therefore, GRMZM2G089713 should be the best candidate gene for the Sh1* locus.

To confirm GRMZM2G089713 is the causative gene for the sh1* phenotype, an allelism test was performed by crossing sh1* with uFmu-02854, a UniformMu insertion mutant of GRMZM2G089713 with W22 genetic background (Fig. 2c). The uFmu-02854 (homozygous) was crossed to sh1* (heterozygous), and the F1 ears displayed a 1:1 segregation of WT (+/uFmu-02854) and shrunken (sh1*/uFmu-02854) phenotype (Fig. 2d), indicating the uFmu-02854 cannot complement sh1*. Therefore, GRMZM2G089713 was the causative gene for the sh1* mutation, and encoded a sucrose synthase SH1 [28]. To further investigate the effect of a single base mutation of *SH1* on its enzymatic activity in the sh1* mutant, we tested the sucrose synthase activity of the endosperm of 16 DAP kernels of sh1*, uFmu-02854, sh1*/uFmu-02854 as well as WT controls Z58 and W22. The results showed that sucrose synthase activity decreased by approximately 91–93% in the endosperm of sh1*, uFmu-02854 and sh1*/uFmu-02854, compared with WT (Fig. 2e). Therefore, we believed that the mutation in sh1* caused its severe loss of *SH1* function, even inactivation.

### *SH1* displayed a close phylogenetic relationship with *SUS1* whereas distinct tissue-specific expression patterns

**Fig. 3:**
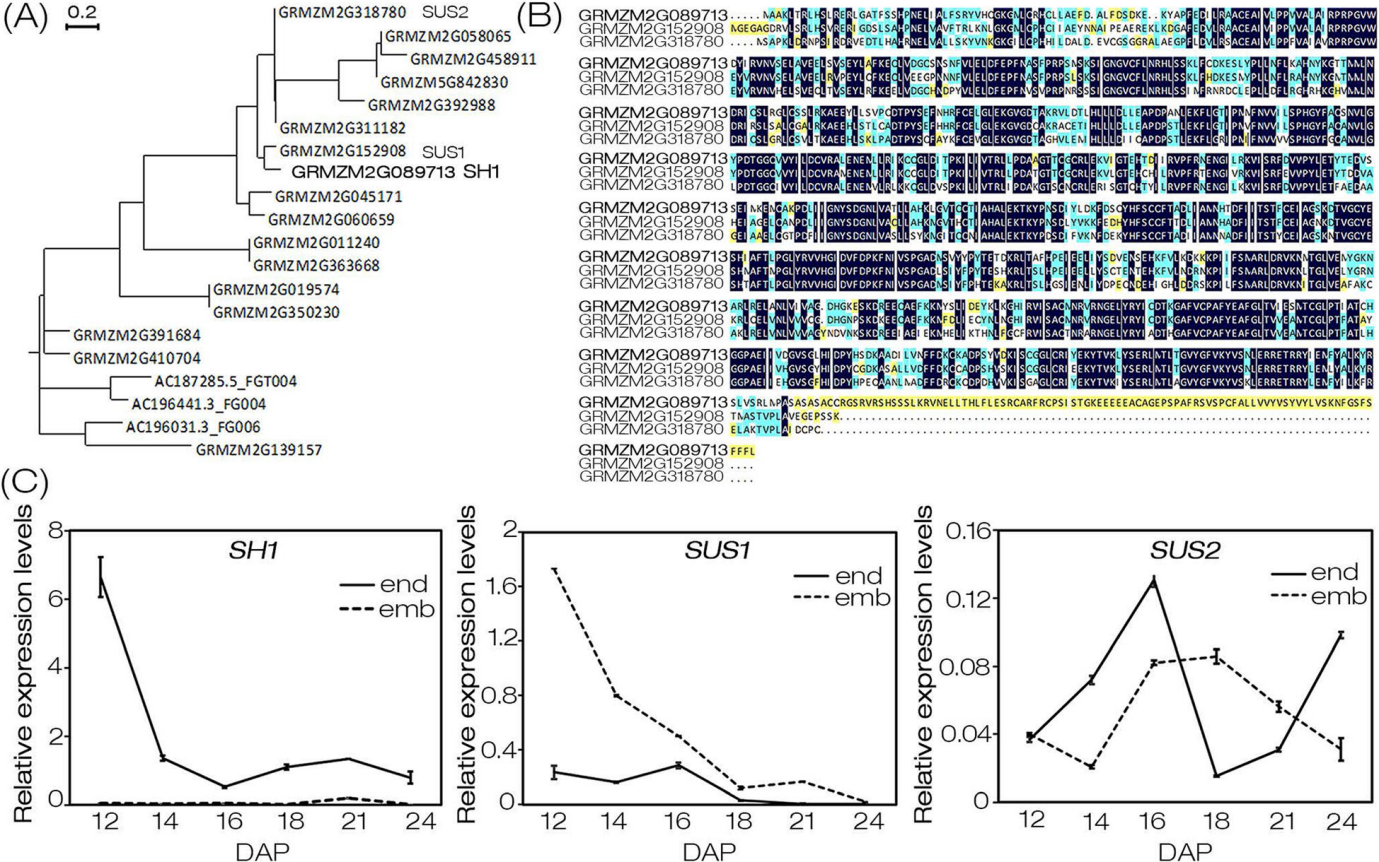
Phylogenetic analysis of SH1 and expression analysis of *SH1*, *SUS1* and *SUS2*. **a** Phylogenetic relationship of the sucrose synthase family in maize. Bar = 0.2 substitutions per site. **b** Multiple sequence alignment of SH1, SUS1 and SUS2. **c** Time-course expression pattern of *SH1*, *SUS1* and *SUS2* in embryo (dotted line) and endosperm (solid line). Average values from three biological replicates were shown. The error bars were used to represent SD

To investigate the phylogenetic relationship of SH1 and its 19 homologs in maize, we constructed a phylogenetic tree based on their amino acids sequences (Fig. 3a). The result showed that SH1 was highly close to SUS1 (GRMZM2G152908). Multiple sequence alignment of SH1, SUS1 and SUS2 (GRMZM2G318780), three sucrose synthases had been identified in maize so far [26, 27], revealing the high identity and conservation of their amino acids sequences, especially SH1 and SUS1 (Fig. 3b). These results suggested that the two of them, especially SH1 and SUS1, might share similar biological functions. However, *SUS1* and *SUS2* were not able to complement the functional deletion mutation of *SH1* in sh1*. To investigate this reason, time-course expression patterns analyses of *SH1*, *SUS1* and *SUS2* in embryo and endosperm of maize were determined. Our results indicated that *SH1* was primarily expressed in endosperm while the *SUS1* transcripts mainly accumulated in the embryo. *SUS2* was expressed in both embryo and endosperm in a different expression trend from with *SH1* and *SUS1*, and its expression levels in the embryo and endosperm were around 1/10 or less than that of *SH1* (Fig. 3c). In addition, previous studies had shown that SH1 and SUS2 displayed different subcellular localizations [9, 26, 27]. These results suggested that their differential expression might account for the inability of *SUS1* and *SUS2* to complement *SH1* function.

### The distribution of SH1 was mostly associated with membranes

**Fig. 4:**
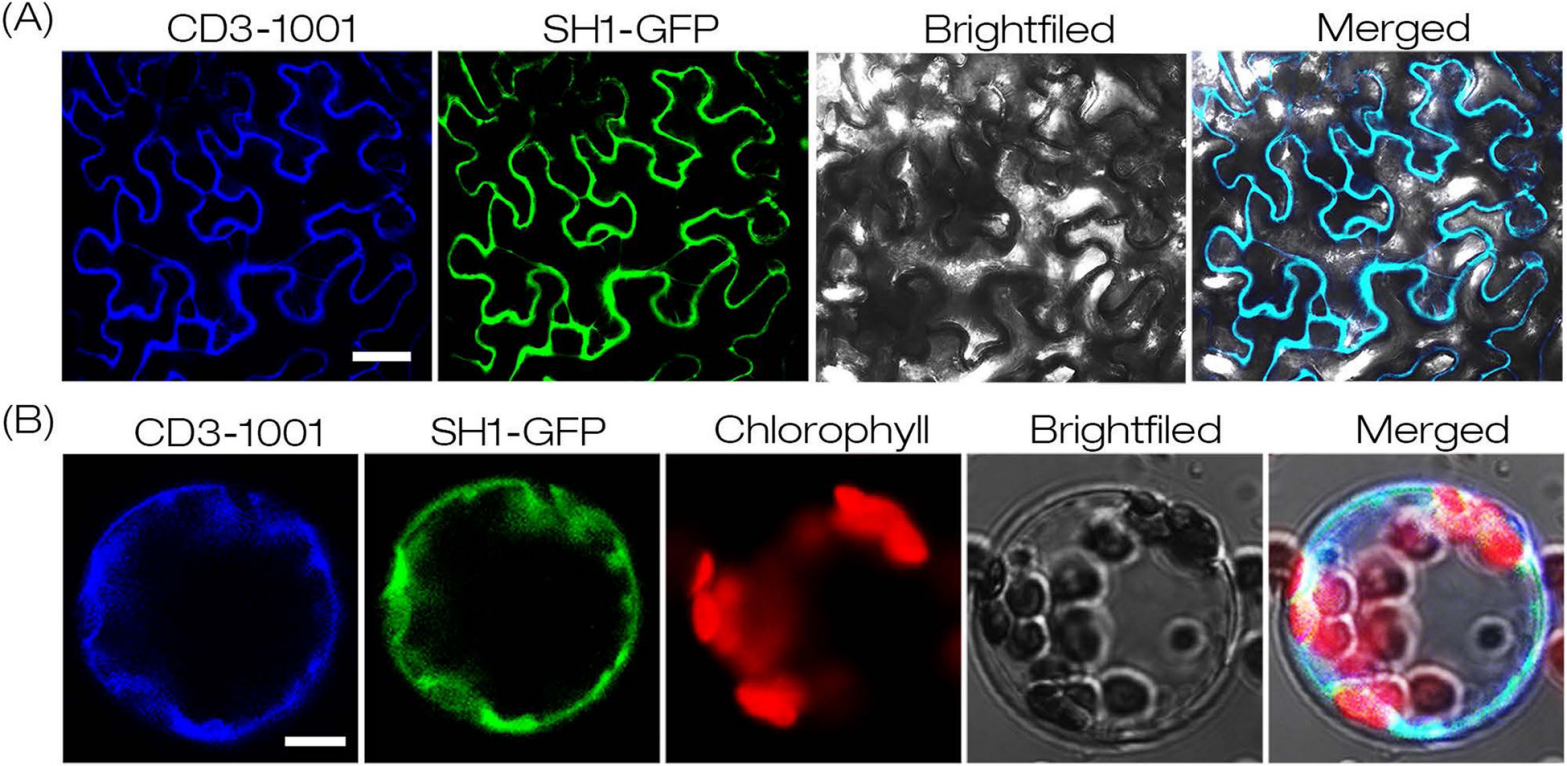
Subcellular localization of SH1. **a** Fluorescence signal observation of tobacco leaves transiently co-expressing *35S::SH1:GFP* and CD3–1001 (Positive control). Bar =20 μm. **b** Fluorescence signal of protoplast transiently co-expressing *35S::SH1:GFP* and CD3–1001. Bar = 5 μm. Bright field and merged of those signals in *Nicotiana benthamiana epidermal* cells or protoplasts were compared

Subbaiah and Sachs (2001) found that SUSs were mainly distributed in the soluble fraction, and only a few of them were membrane-associated revealed by protein separation and immunoblot analysis using non-specific antibody [9]. Duncan et al. (2006) showed that SH1 was associated with membranes, but it was primarily distributed in soluble fraction by protein extraction and immunoblot analysis using isoform-specific antibody [26]. The accuracy of these results largely depended on protein extraction process and antibody specificity. To clarify the localization of SH1, we constructed the *35S::SH1:GFP* plasmid and introduced it together with a membrane protein marker vector CD3–1001 (positive control) into tobacco epidermal cells (Fig. 4a) and protoplasts (Fig. 4b). Laser confocal microscopy revealed that the GFP signals displayed overlapping pattern with the membrane protein marker. This result indicated that the distribution of SH1 may be mostly associated with membranes.

### sh1* affected the balance of carbon metabolism and the maintenance of osmolality during kernel development

**Fig. 5:**
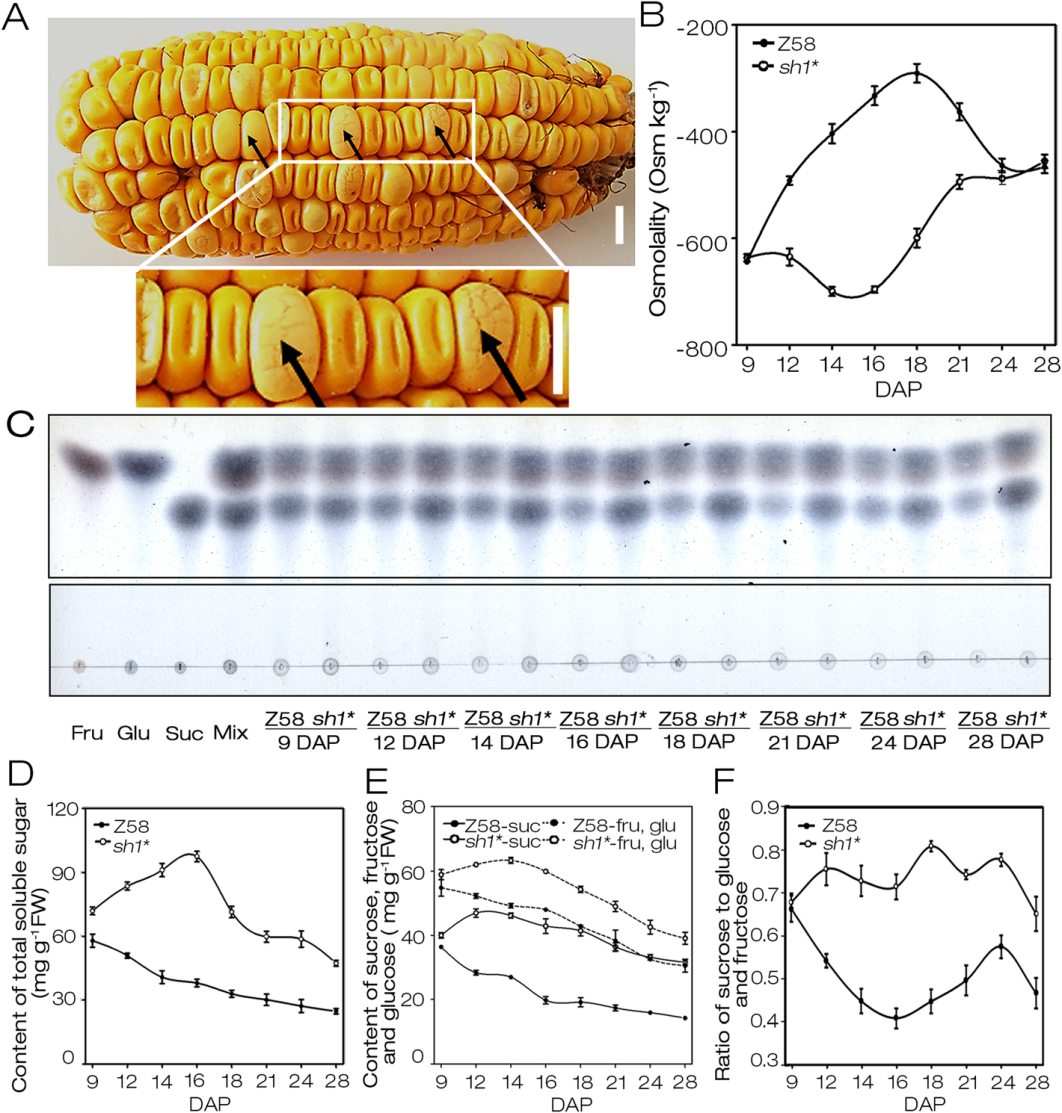
Osmolality and soluble sugar contents in sh1*. **a** A freshly harvested F2 ear of W64A × sh1*. The black arrow identified the sh1* kernels, Bar = 0.5 cm. **b** Comparison of osmolality in Z58 and sh1* kernels. **c** TLC analyses of total soluble sugars from Z58 and sh1* kernels. Fru, Glu and Suc represented fructose, glucose and sucrose, respectively, Mix was a mixture of them. The two groups of gels cropped from different parts of the same gel which is presented in additional Fig. S2 and the cropped areas are labelled using black borders. **d** Comparison of soluble sugar content in Z58 and sh1* kernels. **e** Sucrose, glucose and fructose contents in Z58 and sh1* kernels. **f** Ratio of sucrose to glucose and fructose in kernels of Z58 and sh1*. All experiments mentioned in this picture were performed with three biological replicates and error bars represent SD

When harvesting F2 ears of W64A × sh1*, we found that mutant kernels (sh1*/ sh1*) were swollen extremely and appeared to be bursting (Fig. 5a). This phenotype suggested that the mutant kernels might possess a higher osmotic potential, which drives water absorption in kernels. To clarify the assumption, the osmotic potential of sh1* and Z58 kernels at 9, 12, 14, 16, 18, 21, 24, 28 DAP were detected. Except the kernels at 9 and 28 DAP, the sh1* kernels had a significantly higher osmotic potential than that of Z58. In particular, the osmolality in sh1* kernels at 14, 16 and 18 DAP were more than twice of that in Z58 (Fig. 5b). These results were consistent with that the sh1* kernels had less dry matter accumulation but higher fresh weight than those of Z58 (Fig. 1g).

As a sucrose synthase, SH1 is mainly responsible for the degradation of sucrose. The loss-of-function of *SH1* may affect the composition and content of carbohydrates, leading to a change in osmotic potential. Our results showed that the total soluble sugars in sh1* kernels at 9, 12, 14, 16, 18, 21, 24 and 28 DAP were significantly higher than that of Z58, especially for sh1* kernels of 14 and 16 DAP, even more than 2.5-fold soluble sugar content of that of Z58 kernels (Fig. 5c, d). These results were consistent with the differences of osmotic potential in kernels between sh1* and Z58 (Fig. 5b). Therefore, the dramatical increase of total soluble sugars in developing kernels caused by *SH1* mutation may be the main reason for the enhanced osmotic potential of sh1* kernels. Moreover, the total soluble sugars content in Z58 kernels exhibited a continuous downward trend since 9 DAP, consistent with the rising starch content (Figs. 1j, 5d). However, the total soluble sugars content in sh1* kernels of 9, 12, 14 and 16 DAP presented a sustained increase, consistent with slow starch accumulation compared to Z58 (Figs. 1j, 5d). While soluble sugars content in sh1* kernels was gradually reduced during 16 to 28 DAP, starch synthesis was markedly increased (Figs. 1j, 5d).

To further analyze the differences in soluble sugar components of kernels between sh1* and Z58, the fructose, sucrose and glucose extracted from sh1* and Z58 were assayed by thin layer chromatography (TLC; Fig. 5c). The contents of sucrose, fructose and glucose, and the ratios of sucrose to glucose and fructose in sh1* kernels were significantly higher than that of Z58, except for the kernels of 9 DAP (Fig. 5e, f). Hence, sh1* affected the balance of carbon metabolism and the distribution of carbohydrates in developing maize seeds, and resulted in significant changes in osmotic potential.

### sh1* affected the transcription of genes involved in sugar metabolism and starch synthesis

**Fig. 6:**
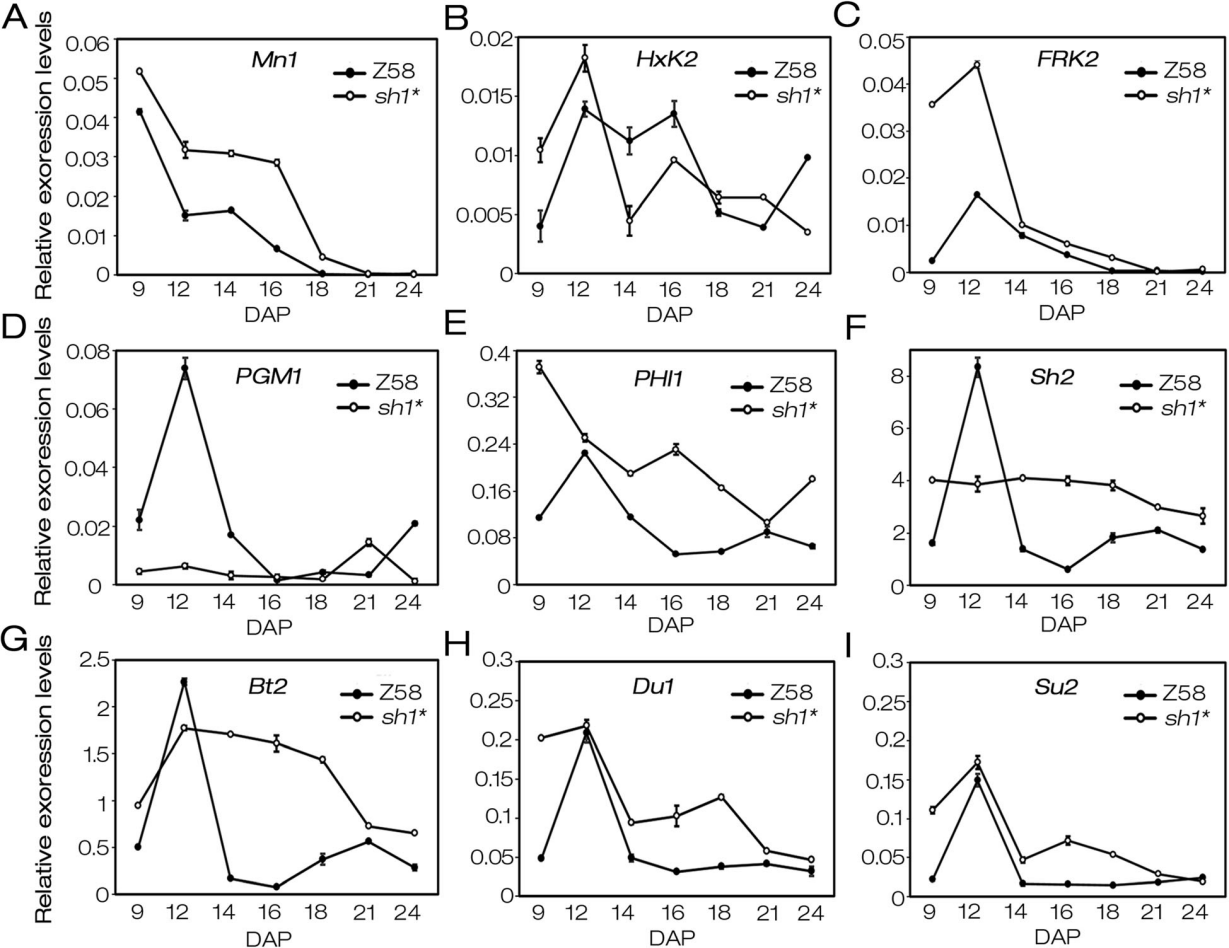
Expression analysis of genes involved in carbon catabolism and starch synthesis. **a-i***Mn1*: cell wall invertase MINIATURE1; *HxK2*: hexokinase 2; *FRK2*: fructokinase 2; *PGM1*: phosphoglucomutase; *PHI1*: phosphohexose isomerase; *Sh2*: shrunken2; *Bt2*: brittle endosperm2; *Du1*: dull endosperm1; *Su2*: sugary2. Ubiquitin (UBQ, GRMZM2G409726) was used as an internal reference. Three times of biological replicates were performed for each gene, and each experiment contained three technical replicates. Values were the means with SD

The expression of carbon metabolism related genes in endosperm of sh1*and Z58 at different developmental stages were detected by real-time quantitative PCR (qRT-PCR; Fig. 6). These genes included *miniature seed1* (*Mn1*, GRMZM2G119689), *hexokinase 2* (*HxK2*, GRMZM2G432801), *fructokinase 2* (*FRK2*, GRMZM2G051677), *phosphoglucomutase* (*PGM1*, GRMZM2G023289), *phosphohexose isomerase* (*PHI1*, GRMZM2G065083), *shrunken 2* (*Sh2*, GRMZM2G429899), *brittle endosperm 2* (*Bt2*, GRMZM2G068506), *dull endosperm 1* (*Du1*, GRMZM2G141399) and *sugary 2* (*Su2*, GRMZM2G348551).

Sucrose degradation and utilization in endosperm mainly depends on two pathways. When functions of *SH1*, the key gene of one of the two pathways, was lost, *Mn1* encoding a key enzyme in another pathway may be up-regulated, as a result of feedback adjustment of sucrose concentration. To clarify this assumption, the expressions of *Mn1* in endosperm of sh1* and Z58 at different developmental stages were detected. These results showed that the expression level of *Mn1* in sh1* was significantly higher than that of Z58 at the same stage (Fig. 6a). As two mediators for coupling sucrose breakdown with hexose phosphate pool by *Mn1*-mediated pathway, *HxK2* and *FRK2* had comparable or higher expression levels in sh1* mutant compared with Z58 (Fig. 6b, c). These not only helped to compensate for the loss of SH1-mediated sucrose degradation pathway in sh1*, but also contributed to the accumulation of hexose phosphate pools, the main precursors for downstream carbon metabolic pathways. As the three main components of the hexose phosphate pools, the conversions of Glc-6-P, Fru-6-P and Glc-1-P are mainly coordinated by *PHI1* and *PGM1*. Compared with Z58, the expression level of *PHI1* was significantly up-regulated in sh1*, while *PGM1* dramatically down-regulated (Fig. 6d, e). PHI1 is responsible for catalyzing the conversion between Glc-6-P and Fru-6-P. The up-regulated expression of *PHI1* in sh1* facilitated the coordinated transformation between Glc-6-P and Fru-6-P. PGM1 catalyzes the transformation between glucose phosphate isomers, and its dramatic down-regulation will be unfavorable to the conversion Glc-6-P to Glc-1-P (a direct precursor for starch synthesis). The lack of Glc-1-P may induce the expression of starch synthesis-related genes, as a result of feedback regulation. To clarify this assumption, expression levels of several key genes involved in starch synthesis, including *Sh2*, *Bt2*, *Du1* and *Su2*, were detected in kernels of sh1* and Z58. These results displayed that, compared with Z58, the expression levels of *Sh2*, *Bt2*, *Du1* and *Su2* were obviously up-regulated during kernels development of sh1*, except for *Sh2* and *Bt2* at 12 DAP (Fig. 6f-g). Therefore, the decrease of starch content in sh1* might be attributed to the lack of substrate for starch synthesis caused by the loss of *SH1*-mediated sucrose degradation pathway.

### The kernels in sh1* had a significantly reduced Glc-1-P content

**Fig. 7:**
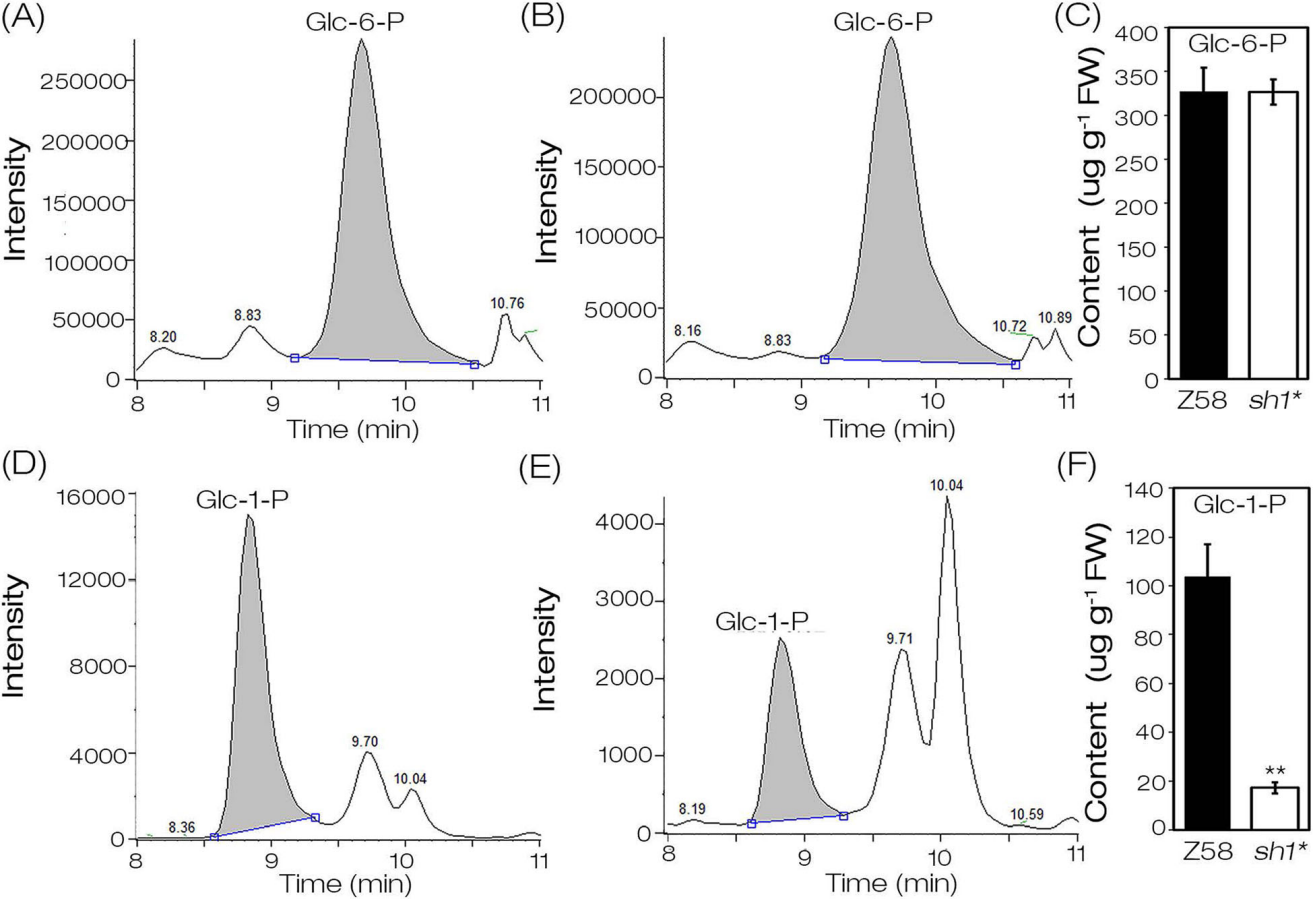
Content of Glucose-1-P and Glucose-6-P in sh1* and Z58 kernels at 12DAP. **a** Chromatogram of examination of glucose-6-P (Glc-6-P) in Z58 kernels. **b** Chromatogram of examination of Glc-6-P in sh1* kernels. **c** The content of Glc-6-P in kernels of Z58 and sh1*. Three biological replicates were performed. Values were means with SD. **d** Chromatogram of examination of glucose-1-P (Glc-1-P) in Z58 kernels. **e** Chromatogram of examination of Glc-1-P in sh1* kernels. **f** The content of Glc-1-P in kernels of Z58 and sh1*. Three biological replicates were performed and values were means with SD (**, P < 0.01, student’s *t* test). Z58, Wild-type control. DAP, day after pollination

The contents of Glc-1-P and Glc-6-P were detected in the 12 DAP kernels of Z58 and sh1* (Fig. 7). The results showed that the contents of Glc-6-P was almost the same between sh1* and Z58 (Fig. 7a-c), whereas compared with Z58, the content of Glc-1-P was greatly reduced in sh1* (Fig. 7d-f). The content of Glc-1-P in kernels of sh1* was 17 μg g^− 1^ FW, which was only about 5.2% of that in Z58 (326 μg g^− 1^ FW), indicating that the substrates deficiency may be an important factor of the decreased starch content in sh1*. Therefore, the functional loss of *SH1* led to more carbon flow to the glycolysis pathway, and less to starch synthesis pathways, resulting in shrunken phenotype of sh1* kernels (Fig. 1a, b).

## Discussion

As the main form of carbohydrates transported from vegetative tissues to the reproductive organs, sucrose are vital for carbon source supply, energy metabolism, synthesis of cellulose and starch and so on. In addition, sucrose and its degradation products (hexoses) also function as signaling molecules to regulate cell fate determination and kernel development by affecting the expression of related genes [2, 29–31]. Hence, the regular sucrose catabolic pathways are essential for normal kernel development. Because of its importance, *SH1* has attracted attentions as a key enzyme for sucrose degradation. The early studies of *SH1* mostly focused on the gene structure [32, 33], the enzyme activity [28, 34, 35], and the interallelic complementation at the *Sh* locus [36]. Recent research has shown that the functional loss of *SH1* led to the decrease of starch accumulation [8, 11]. However, the molecular and physiological regulatory mechanisms are still unclear. In this study, a new allelic mutant (sh1*) of *sh1* was identified and characterized. The mutation of *SH1* in sh1* caused more carbon sources to exist as soluble sugars, and less flow to starch synthesis pathway (Figs. 1j, 5d, 7f). The modulated carbohydrates distribution led to the lack of starch-synthesis substrates and the high osmotic stress in developing endosperm cells in sh1*.

### The significant decrease of G-1-P content caused by the *SH1* null mutation contributed greatly to the reduction of starch synthesis

Previous studies showed that the functional loss of *SH1* led to significant decrease in the starch content and shrunken phenotype of kernels in maize. Chourey et al. (1998) reported that the starch contents in kernels of *sh1* was about 78% of that in WT [8]. Guan et al. (2017) observed the starch content in *sh1-m*, a new allelic mutant of *SH1*, was about 62% of that in WT, but thereof is no any explanation for this result [11]. In this study, time-course detection displayed that the starch in kernels of sh1* were dramatically less accumulated at same stages, except for the kernels at 9 DAP (Fig. 1j). Hence, it is confirmed that *SH1* plays an important role in regulating starch synthesis.

The expression levels of key genes (such as *Sh2* and *Bt2*) related to starch synthesis indicated that they were significantly up-regulated in sh1* (Fig. 6f-i), suggesting the ability of starch synthesis in sh1* may not be weakened. Giroux et al. (1994) reported that the expression of storage product genes, specifically *Bt2* and *Sh2* encoding ADP-glucose pyrophosphorylase subunits, could be induced by elevated sucrose levels [45]. So, the increased expression of starch synthesis related genes may be attributed to the elevated sucrose levels in the sh1* mutant (Fig. 5c, e). These results suggested that the decrease in starch content of sh1* kernels might be not due to the weakened starch synthesis ability.

**Fig. 8:**
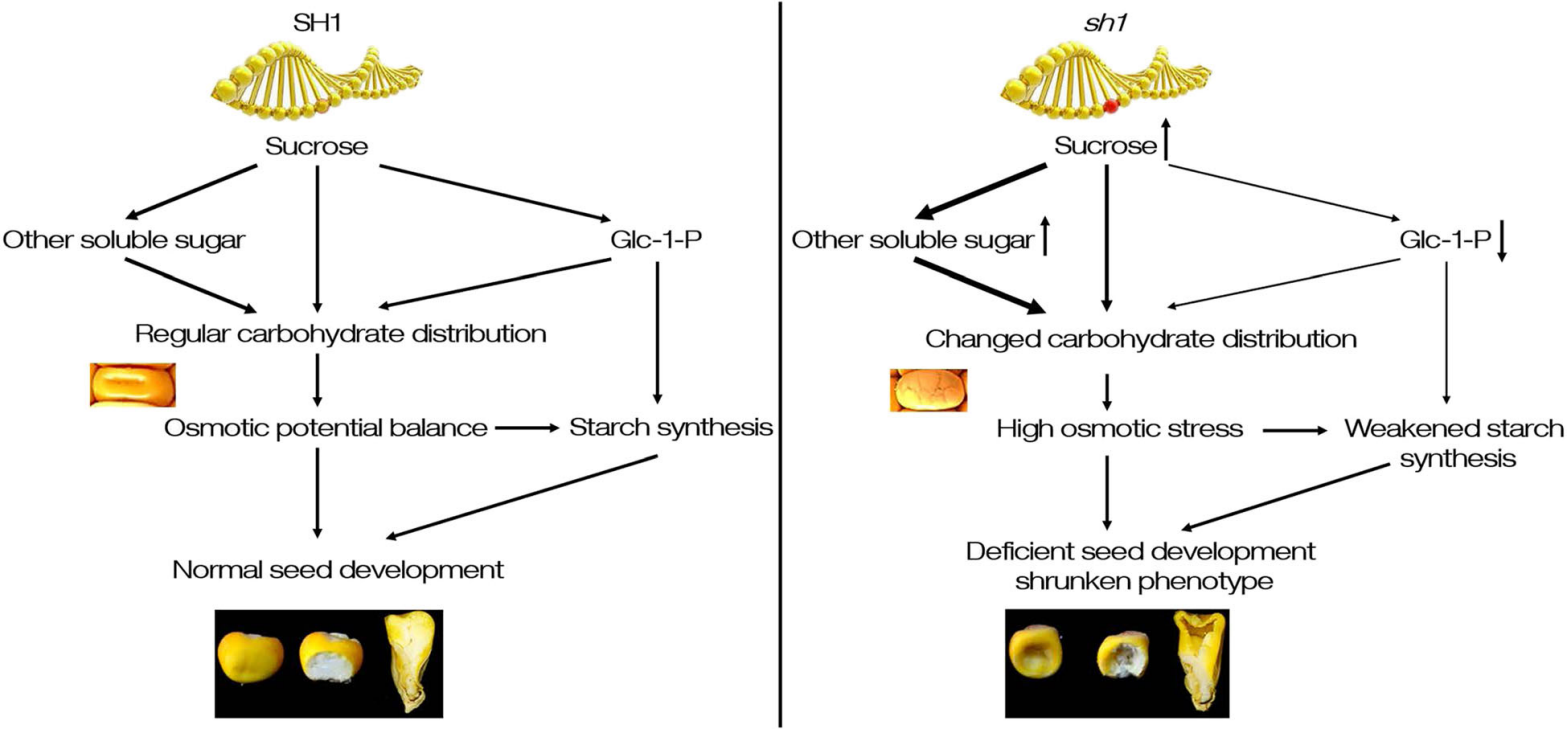
Model of the influence of carbohydrate flow mediated by SH1 on kernel development. The thickness of the arrow indicated the activity of the reaction. Upward arrows behind sucrose and other soluble sugar indicated the increased content, downward arrows behind Glc-1-P indicated decreased content in the case of *SH1* mutation

G-1-P, the direct substrate for starch synthesis, is mainly transformed from the two branches of UDP-Glc and G-6-P. UDP-Glc, as one of the sucrose degradation products catalyzed by SUS, the significant decrease of SUS activity inevitably reduces the production of UDP-Glc, which in turn leads to the reduction of G-1-P converted from UDP-Glc. Although the SUS family has 20 members in the maize genome (Fig. 3a), the loss-of-function of *SH1* leads to the decrease of approximately 90–95% of SUS activity in maize endosperm [28, 34, 36], indicating that *SH1* plays a key role in sucrose degradation of maize endosperm cells. In the study, a single base mutation of *SH1* results in the loss of about 92% of SUS activity in sh1* endosperm (Fig. 2e) and shrunken phenotype (Fig. 1a and b), so sh1* can be considered as a mutant with loss of *SH1* function. Therefore, G-1-P converted from UDP-Glc will be greatly reduced in endosperm of sh1* mutant. In addition, the conversion of G-6-P to G-1-P requires PGM1 catalysis. The null mutation of *SH1* resulted in a significant down-regulation of *PGM1* expression (Fig. 6d), suggesting a rate-limiting flux of G-6-P to G-1-P. Indeed, the content of Glc-1-P in endosperm of sh1* (17 μg g^− 1^ fresh weigh) was only 5.2% of Z58 (326 μg g^− 1^ fresh weigh; Fig. 7d-f). The results indicated that the loss-of-function of *SH1* largely blocked the production of G-1-P, suggesting that *SH1*-encoded sucrose synthase may play critical roles in the supply of substrates for starch synthesis during kernel development. Therefore, the *SH1*-mediated sucrose degradation greatly contributed to kernels development and starch synthesis by regulating the flow of carbohydrates (Fig. 8).

### The lack of SH1-mediated sucrose degradation triggered transcriptional responses distinct from that mediated by *MN1*

Two key enzyme families, INVs and SUSs are involved in sucrose degradation. INVs irreversibly catalyze the breakdown of sucrose into fructose and glucose, whereas SUSs catalyze the conversion of sucrose to fructose and UDP-Glc in a reversible manner [9, 25–27]. In maize, Mn1 and SH1 are widely considered as key enzymes in the INVs and SUSs family, respectively. Both loss-of-function mutants of *Mn1* and *SH1* (*mn1* and *sh1*) had significant kernel development defects and reduced starch content [8, 11, 24, 27]. However, the regulation mechanisms of kernel developmental defects caused by mutation of *Mn1* or *SH1* are significantly different. Chourey et al. (2012) found that *Mn1* mutation resulted in an obvious reduction in fructose and glucose contents, and a significantly reduced ratio of hexose to sucrose [24]. Our results showed that although the functional loss of *SH1* led to an obviously reduced ratio of hexose to sucrose, the contents of fructose, glucose and total soluble sugars in sh1* were significantly higher than that of WT (Fig. 5e, f). Correspondingly, a large number of genes related to carbon metabolism and starch synthesis had significantly differential expression trends between *mn1* and *sh1*. They thought that the hexose-deficiency was the main cause of the metabolic coordinated regulation in *mn1* [24]. In this study, we found that almost all genes involved in carbon metabolism and starch synthesis, including *Mn1*, *Su2, FRK2*, *PHI1*, *Sh2*, *Bt2* and *Du1*, were significantly up-regulated in the endosperm of sh1* (Fig. 6). While *SH1*-mediated sucrose degradation pathway was blocked in the sh1*, the *Mn1*-mediated sucrose degradation pathway was significantly induced to compensate for the sucrose degradation defects caused by *SH1* mutation. It is noticeable that when *Mn1*-mediated sucrose degradation was inhibited in the *mn1* mutant, *SH1*-mediated sucrose degradation would be significantly inhibited [24]. Based on these results, we suggested that *Mn1* and *SH1* might function in different ways during kernel development in maize.

To explore the roles of sucrose degradation and utilization during maize kernel development more clearly and accurately, more efforts are still required. For example, generation of constructing *sh1 mn1* mutants by crossing sh1* and *mn1* may help to understand the functional interactions between INVs and SUSs in details.

## Conclusions

In the study, a novel *SH1* mutant (sh1*) was characterized. Null mutation of *SH1* resulted in defects in kernel development and starch granule formation, including markedly decreased starch content, significantly increased soluble sugar content and osmotic potential. The measurement results of phosphorylated sugars showed that the loss-of-function of *SH1* resulted in a significant decrease of G-1-P content in sh1* endosperm. As a direct source of starch synthesis, the decrease of Glc-1-P may result in a significant reduction in carbohydrates that flow to starch synthesis, ultimately contributing to the defects in starch granule development and reduction of starch content. These results provided new insights into understanding of the roles of *SH1* in starch synthesis and maize kernel development.

## Methods

### Plant materials

The sh1* mutant was separated from EMS induced mutants’ library in Z58 genetic background in our laboratory. The inbred line Z58 is the female parent of the hybrid Zhengdan958 and has been widely used in the maize hybrid breeding in china. The uFmu-02854 mutant was obtained from the Maize Genetics Cooperation stock center. The sh1* was crossed to W64A to produce F1, and F1 self-crossing generates F2 mapping population. All plants were cultivated in the field on the outskirts of Jinan, Shandong Province, China. Kernels were harvested at 9, 12, 14, 16, 18, 21, 24 and 28 DAP.

### Paraffin sections

For anatomical observation, kernels were sectioned based on paraffin embedding as previously described [3]. Briefly, kernels were fixed in FAA buffer (glacial acetic acid: formaldehyde: 50% ethanol, 1: 1: 18) at 4 °C for 24 h, and dehydrated in different concentrations of ethanol. Subsequently, the samples were embedded in paraffin, cut into 8 μm-thick slices, and stained with basic fuchsin and observed using an Olympus SZ61 microscope.

### Scanning Electron microscopy

For starch granule observation, the naturally ruptured endosperm from dry mature kernels were coated with gold and then observed using scanning electron microscopy (FEI Quanta250 FEG).

### Map-based cloning

To map the sh1* locus, 2400 individuals from a W64A × sh1* F2 segregating population were used. Preliminary mapping was performed using molecular markers distributed on maize chromosome 9. The molecular markers listed in Additional file 1 (Table S1) were used for fine-mapping and eventually the sh1* locus was localized to a 97.12 kb region.

The genomic DNA of sh1* and Z58 were extracted. The nucleotide sequence of predicted genes within this region was amplified from the sh1* and Z58 genomic DNA using TransStart^Ɍ^ FastPfu DNA polymerase (Transgen Biotech), and aligned after sequencing by Shanghai Boshang Biotechnology Co., Ltd.

### Phylogenetic analysis of SUSs

The amino acid sequences of SUSs were download from MaizeGDB (https://maizegdb.org/). The phylogenetic analysis of them were performed with the maximum likelihood according to [7]. Phylogenetic tree was constructed using MEGA7.0 Amino acid sequences alignment of GRMZM2G089713 (SH1), GRMZM2G152980 (SUS1) and GRMZM2G318780 (SUS2) were performed using DNAMAN 7.0 (Lynnon Corporation, USA).

### Subcellular localization

The CDS sequence of *SH1* was ligated into pEarleyGate 103 using the Gateway TOPO cloning kit to generate a *35S::SH1:GFP* fusion construct. Then, this vector was introduced into 6-weeks-old *Nicotiana benthamiana* leaves by *Agrobacterium* impregnation. After 48 h, the *Nicotiana benthamiana* epidermis was examined with a confocal microscope (Zeiss LSM700). For protoplast transformation, the plasmid of *35S::SH1:GFP* was introduced into protoplast isolated from 2-weeks-old *Nicotiana benthamiana* leaves via PEG-mediated transformation. After 18 h, protoplasts were examined as described above. CD3–1001 (CFP) was used as a membrane protein marker and mixed with bacterial solution 1:1 to infect tobacco leaves and protoplast.

### The determination of osmolality

The sh1* and Z58 kernels at different developmental stages were homogenized and centrifuged separately, then pipetting 20 μl of supernatant to use for the determination of osmolality by the Fiske Micro-Osmometer (Model 210). Three biological replicates were done.

### The measurement of soluble sugar and starch content

The total soluble sugars of sh1* and Z58 kernels at different developmental stages were extracted three times in 80% ethanol at 80 °C for 30 min after thoroughly triturated, respectively. For the quantification of soluble sugars, a constant volume of those supernatants were maintain in the wake of 80% ethanol added, then extracts were diluted in distilled water and measured with the anthranone-sulfuric acid reagent at 620 nm as described previously [37, 38]. The fructose, glucose and sucrose were differentiated by thin layer chromatography (TLC) based on the method by Li et al. (2008) [20]. The total soluble sugars extracted from kernels in same fresh weight (0.2 mg) were used for TLC analysis. TLC image were further analyzed at 390 nm wavelength using a Cs-930 dual-wavelength chromatoscanner (Shimadzu) to semi-quantitated the content of sucrose, glucose and fructose. The pellets derived from total soluble sugars extraction were used for starch measurement. After removing the ethanol, those pellets were boiled with distilled water for 10 min, then cooled down to room temperature, and decomposed those samples with HClO_4_ for 15 min. The products of hydrolyzation were examined at 620 nm as described above. Formula below was for starch content calculation: Starch content (%) = G × 0.9 FW^− 1^ × 100%. (G: content of glucose in these samples, FW: fresh weight of these kernels).

### RNA extraction and gene expression assays

The sh1* and Z58 kernels at different developmental stages were carefully removed the seed coat and separated the embryo and endosperm. The total RNA of embryos and endosperms were extracted using Trizol reagent (Sangon, China) as product manual. The 2 μg total RNA of each sample was used as the template of cDNA synthesis that was carried out using a PrimeScript TM RT reagent Kit with genome DNA Eraser (Takara, China). SYBR Green PCR master mix kit (Takara, China) and Light Cycler®96 PCR machine (Roche) were used for real-time quantitative PCR (qRT-PCR) examination. Ubiquitin (UBQ, GRMZM2G409726) was used as an internal reference. Primers used in qRT-PCR were listed in Additional file 2 (Table S2). Three biological replicates were done for gene expression analysis.

### The measurement of Glc-1-P and Glc-6-P contents

Glc-1-P and Glc-6-P contents were determined by the Wuhan Greensword Creation Technology Co. Ltd., using UHPLC-MS/MS according to the previous method [39]. Twelve DAP kernels of Z58 and sh1* were ground to power in the presence of liquid nitrogen. 5 μl (25 ng) [^13^C_6_] G-6-P at 5 μg ml^− 1^ was added to the samples as an internal reference (I.S.) for accurate quantification. 250 μl ice-cold mixture of chloroform and acetonitrile (3:7, v v^− 1^), was added to Z58 and sh1* samples, respectively. After thoroughly mixing, these samples were incubated at − 20 °C, with intermittent mixing, for 2 h. Next, 200 μl cold water in ice bath was added to the reaction solution, then vortexed for 3 min. The upper aqueous acetonitrile phase was separated and transferred to new 1.5 ml centrifuge tubes after centrifugation (10,000×g, 4 °C) for 5 min. The chloroform phase was extracted once more and mixed with the aqueous acetonitrile phases. The residue, produced by evaporating the aqueous acetonitrile phases under mild nitrogen stream, was re-dissolved in 200 μl borate buffer (50 mM, pH 6.8), and purified with C18 SPE cartridge (50 mg of C18 sorbent), then washed with the borate buffer (50 mM, pH 6.8, 100 μl). The elutes were collected, and added with 180 μl of 8-DMQ solution (0.014 M) into it. The reaction was carried out during the vigorously shaking at 25 °C for 40 min and centrifuged 3 min at 10,000×g. The solution (2.5 μl) was analyzed by ultra-high performance liquid chromatography-electron spray ionization-tandem mass spectrometry (UHPLC-ESI-MS/MS).

### The measurement of sucrose synthase activity

A mixture of equal weight of endosperm at 16 DAP and chilled 0.01 U tris-maleate buffer (pH 7.0) was homogenized, and then centrifuge at 30,000 g for 20 min. The supernatants were used as the enzyme sources for the routine assays. The activity of sucrose synthase was assayed according to the description by Tsai et al. (1970) [40]. The 0.55 ml reaction mixture contained 30 μmol of HEPES buffer (pH 8.0), 2.5 μmol MgSO_4_, 2 μmol fructose, 1 μmol UDP-glucose, and 10 μl crude preparation. After the reaction at 30 °C for 30 min, NaOH was added to stop the reaction. Boiling water for 10 min, cooling and adding 1.5 ml of 30% hydrochloric acid and 0.5 ml of 0.1% phloroglucinol, after shaking for 10 min, the content of sucrose was determined at 480 nm wavelength after cooling, the standard curve of sucrose was made at the same time. Finally, the activity of sucrose synthase in the sample was calculated. Three biological replicates were done.

## Supplementary information

**Additional file 1: Table S1.** The list of molecular markers and primers used in gene cloning.

**Additional file 2: Table S2.** The list of primers used in qRT-PCR reaction.

**Additional file 3: Fig. S1.** The sequencing data of GRMZM2G089713 in Z58 and sh1*.

**Additional file 4: Fig. S2.** Full size, unedited gel used for Fig. 5c in the main text.

## Data Availability

All data generated or analysed during this study are included in this published article and its additional information files. The sequencing data of GRMZM2G089713 in Z58 and *sh1** see additional Fig. S1. All plant materials were obtained from Shandong University, Qingdao, China.

## Abbreviations

*Bt2*: *brittle endosperm 2*
BETL: Basal endosperm transfer layer;
DAP: Days after pollination
*Du1*: Dull endosperm 1
EMS: Ethyl methanesulfonate
*FRK2*: F*ructokinase 2*
Fru-6-P: Fructose-6-phosphate
Glc-1-P: Glucose-1-phosphate
Glc-6-P: Glucose-6-phosphate
*HxK2*: *Hexokinase 2*
INV: Invertase
*Mn1*: *MINIATURE1*
ORF: Open reading frame
*PGM1*: *Phosphoglucomutase*
*PHI1*: *Phosphohexose isomerase*
qRT-PCR: Real-time quantitative PCR
*Sh2*: *Shrunken 2*
*Su2*: *Sugary 2*
SUS: Sucrose synthase
SUTs: Sucrose transporters
TLC: Thin layer chromatography
UDP-Glc: Uridine-diphosphoglucose
WT: Wild-type.
