# Supplementary material for: SH1-dependent maize seed development and starch synthesis via modulating carbohydrate flow and osmotic potential balance
Source: BMC Plant Biol. 2020 Jun 8;20:264. doi: 10.1186/s12870-020-02478-1 (PMC7282075; doi:10.1186/s12870-020-02478-1)
Supplement: Supplementary file 1 — Additional file 1: Table S1. The list of molecular markers and primers used in gene cloning. [file 12870_2020_2478_MOESM1_ESM.pdf]

Table S1 The list of molecular markers and primers used in gene cloning.

Name	Forward (5'→3')	Reverse (5'→3')
ZM0031	ATCGCGACGAGTTAATTCAAACAT	ACGATGTCTTCAGTGTGACACCA
ZM0037	ATGCACATGCAGTTCCTTGGTTAT	GAAAATGAAGAATAGGAGACATTGTTGC
ZM0042	GTCGTCGATCATAGGGAACG	GTGTCCGCAGTTCGCTCA
ZM0047	GCCCTACTTTCTATTTCGGTG	TGAATAATGAATTAGCCCTTG
ZM0055	TGTGCATGGTGTTCAAGTGATT	ATCCTGTCGTTCGTCCTTCA
ZM0056	ACAGGTCAGAGGGAGTAGCATT	CCTTCCCCAGACCTCACA
ZM0059	CTACGTGGAAGCCATTCGAGGTCAG	ATACTACCCTACCTCCGGCCAACAA
ZM0062	AATCTCAACAAGTGACCAAATGCG	CGGTAGGCAGCGTTGTGAAG
ZM0064	CCAGGCGGGTAGATAGC	GGAGATGGAGACGATGATGT
SH1	ATGGCTGCCAAGCTGAC	ATCGAAGGACAGCGGAAC
