# Supplementary material for: SH1-dependent maize seed development and starch synthesis via modulating carbohydrate flow and osmotic potential balance
Source: BMC Plant Biol. 2020 Jun 8;20:264. doi: 10.1186/s12870-020-02478-1 (PMC7282075; doi:10.1186/s12870-020-02478-1)
Supplement: Supplementary file 2 — Additional file 2: Table S2. The list of primers used in qRT-PCR reaction. [file 12870_2020_2478_MOESM2_ESM.pdf]

Table S2 The list of primers used in real-time quantitative PCR reactions

Gene name	Accession number	Primer sequences (5'→3')
UBQ	GRMZM2G409726	FP-1: CTGGTGCCCTCTCTCCATATGG
		RP-1: CAACACTGACACGACTCATGACA
SH1	GRMZM2G089713	FP-2: CACGACGATGATGTTGAATGAC
		RP-2: CGAGAAGCAAGTGGAGTGTGTC
SUS1	GRMZM2G152908	FP-3: CCTGTCCATCTACTTCCCGTA
		RP-3: GATTGGCTTGTTCCTGTCGT
SUS2	GRMZM2G318780	FP-4: ACTTTCCACATACCGAGAAGGCCA
		RP-4: AAGGTTTACCAGCTCCCTCAGCTT
Mn1	GRMZM2G119689	FP-5: ACGGACATCTCGAACGGCAAGATA
		RP-5: CGTTCATGACCGGCTTCTTCATCT
HxK2	GRMZM2G432801	FP-6: GTACCAAGCGGTGGCGGTAAAAT
		RP-6: CTAGTCACTCGCGCCATACTGA
FRK2	GRMZM2G051677	FP-7: ACAAGGGATGCAGGTACTTCACCA
		RP-7: TCTCCTCGTTGTGGAAGATGGAGT
PGM1	GRMZM2G023289	FP-8: ATGATCCATCCTCTTGTGCTTT
		RP-8: CTTCTTCGTCACGGTGAAGAG
PHI1	GRMZM2G065083	FP-9: CAACTTTCCTTTGAGACAGGTG
		RP-9: CTGCTGACTTTTAACAACACCA
Sh2	GRMZM2G429899	FP-10: CGACGCAATTGGACAAGTGCAAGA
		RP-10: AGTTTCATAGGTGTCCGCTCCCAT
Bt2	GRMZM2G068506	FP-11: TATTACCGTTGCTGCCCTACCGAT
		RP-11: GCATTTCCTTTGCCCTCACGTCAT
Su2	GRMZM2G348551	FP-12: GGGGAAGTAGGCAGGAAATCAT
		RP-12: CAGATAAACAGGCAGGAGTGC
Du1	GRMZM2G141399	FP-13: GCTTCTTCTGTCGTTCTGCTCT
		RP-13: GCGTAGTTTTCCTTGTGTAGCC
