# Supplementary material for: SH1-dependent maize seed development and starch synthesis via modulating carbohydrate flow and osmotic potential balance
Source: BMC Plant Biol. 2020 Jun 8;20:264. doi: 10.1186/s12870-020-02478-1 (PMC7282075; doi:10.1186/s12870-020-02478-1)
Supplement: Supplementary file 3 — Additional file 3: Fig. S1. The sequencing data of GRMZM2G089713 in Z58 and sh1*. [file 12870_2020_2478_MOESM3_ESM.pdf]

>Z58

ATGGCTGCCAAGCTGACTCGCCTCCACAGTCTTCGCGAACGCCTTGGTGCCACCTTCTCCTCCCATCC
CAATGAACTGATAGCACTCTTTTCCAGGTATGTTCACCAGGGCAAGGGAATGCTTCAGCGCCATCAGC
TGCTTGCGGAGTTTGATGCCCTGTTTGATAGTGACAAGGAGAAGTATGCACCCTTTGAAGACATTCTTC
GTGCTGCTCAGGAAGCAATTGTGCTCCCCCCATGGGTTGCACTTGCTATCAGGCCAAGGCCTGGTGTC
TGGGATTACATTCGGGTGAATGTAAGTGAGCTGGCTGTGGAGGAGCTGAGTGTTTCTGAGTACTTGGC
ATTCAAGGAACAGCTGGTGGATGGACAATCCAACAGCAACTTTGTGCTTGAGCTTGATTTTGAGCCCT
TCAATGCCTCCTTTCCTCGTCCTTCCATGTCGAAGTCCATCGGAAATGGAGTGCAATTCCTTAACCGAC
ACCTGTCGTCCAAGTTGTTCCAGGACAAGGAGAGTTTGTACCCCTTGCTGAACTTCCTCAAGGCTCAT
AACTACAAGGGCACGACGATGATGTTGAATGACAGAATCCAAAGCCTTCGTGGTCTCCAATCATCCCT
GAGAAAGGCAGAGGAGTATCTACTGAGTGTTCCTCAAGACACTCCCTACTCGGAGTTCAACCATAGGT
TCCAAGAGCTTGGCTTGGAGAAGGGTTGGGGTGACACTGCGAAGCGTGTACTCGACACACTCCACTT
GCTTCTCGACCTTCTGGAGGCCCCTGATCCTGCCAACTTGGAGAAGTTCCTTGGAACTATACCAATGAT
GTTCAACGTTGTTATCCTGTCTCCTCATGGCTACTTCGCCCAGTCCAATGTGCTTGGATACCCTGACACT
GGCGGTCAGGTTGTGTACATTCTGGATCAGGTCCGTGCTTTGGAGAATGAGATGCTTCTGAGGATTAA
GCAGCAAGGCCTTGATATCACTCCGAAGATCCTCATTGTTACCAGGCTGTTGCCTGATGCTGCTGGGAC
TACGTGCGGTCAGCGGCTGGAGAAGGTCATTGGTACTGAGCACACAGACATCATTCGCGTTCCCTTCA
GAAATGAGAATGGCATCCTCCGCAAGTGGATCTCTCGTTTTGATGTCTGGCCATACCTGGAGACATACA
CTGAGGATGTTTCCAGTGAAATAATGAAAGAAATGCAGGCCAAGCCTGACCTTATCATTGGCAACTAC
AGCGATGGCAACCTAGTCGCCACTCTGCTCGCGCACAAGTTGGGAGTCACTCAGTGTACCATCGCTCA
TGCCTTGGAGAAAACCAAATACCCCAACTCGGACATATACTTGGACAAATTCGACAGCCAGTACCACT
TCTCTTGCCAGTTCACAGCTGACCTTATTGCCATGAACCACACTGATTTCATCATCACCAGCACATTCC
AAGAAATCGCGGGAAGCAAGGACACCGTGGGGCAGTACGAGTCCCACATCGCGTTCACTCTTCCTGG
GCTCTACCGTGTCGTCCATGGCATCGATGTTTTCGATCCCAAGTTCAACATTGTCTCTCCTGGAGCAGA
CATGAGTGTTTACTACCCGTATACGGAAACCGACAAGAGACTCACTGCCTTCCATCCTGAAATCGAGG
AGCTCATCTACAGCGACGTCGAGAATTCCGAGCACAAGTTCGTGCTGAAGGACAAGAAGAAGCCGAT
CATCTTCTCGATGGCGCGTCTCGACCGCGTGAAGAACATGACAGGCCTGGTCGAGATGTACGGCAAGA
ACGCGCGCCTGAGGGAGCTGGCGAACCTCGTGATCGTTGCCGGCGACCACGGCAAGGAGTCCAAGG
ACAAGGAGGAGCAGGCGGAGTTCAAGAAGATGTACAGCCTCATCGACGAGTACAAGTTGAAGGGCC
ATATCCGGTGGATCTCGGCGCAGATGAACCGCGTCCGCAACGGGGAGCTGTACCGCTACATTTGCGAT
ACGAAGGGCGCATTCGTGCAGCCTGCGTTCTACGAAGCGTTCGGCCTGACTGTGATCGAGTCCATGAC
GTGCGGTCTGCCAACGATCGCGACCTGCCATGGTGGCCCTGCTGAGATCATCGTGGACGGGGTATCTG
GCCTGCACATTGACCCTTACCACAGCGACAAGGCCGCGGATATCCTGGTCAACTTCTTTGACAAATGC
AAGGCAGATCCGAGCTACTGGGACAAGATCTCACAGGGCGGCCTGCAGAGAATTTATGAGAAGTACA
CCTGGAAGCTCTACTCCGAGAGGCTGATGACCCTGACCGGCGTGTACGGGTTCTGGAAGTACGTGAG
CAACCTGGAGAGGCGCGAGACCCGCCGCTACATCGAGATGTTCTACGCCCTGAAGTACCGTAGCCTGG
CAAGCCAGGTTCCGCTGTCCTTCGATTAG

>sh1*

ATGGCTGCCAAGCTGACTCGCCTCCACAGTCTTCGCGAACGCCTTGGTGCCACCTTCTCCTCCCATCC
CAATGAACTGATAGCACTCTTTTCCAGGTATGTTCACCAGGGCAAGGGAATGCTTCAGCGCCATCAGC
TGCTTGCGGAGTTTGATGCCCTGTTTGATAGTGACAAGGAGAAGTATGCACCCTTTGAAGACATTCTTC
GTGCTGCTCAGGAAGCAATTGTGCTCCCCCCATGGGTTGCACTTGCTATCAGGCCAAGGCCTGGTGTC
TGGGATTACATTCGGGTGAATGTAAGTGAGCTGGCTGTGGAGGAGCTGAGTGTTTCTGAGTACTTGGC
ATTCAAGGAACAGCTGGTGGATGGACAATCCAACAGCAACTTTGTGCTTGAGCTTGATTTTGAGCCCT

TCAATGCCTCCTTTCCTCGTCCTTCCATGTCGAAGTCCATCGGAAATGGAGTGCAATTCCTTAACCGAC
ACCTGTCGTCCAAGTTGTTCCAGGACAAGGAGAGTTTGTACCCCTTGCTGAACTTCCTCAAGGCTCAT
AACTACAAGGGCACGACGATGATGTTGAATGACAGAATCCAAAGCCTTCGTGGTCTCCAATCATCCCT
GAGAAAGGCAGAGGAGTATCTACTGAGTGTTCCTCAAGACACTCCCTACTCGGAGTTCAACCATAGGT
TCCAAGAGCTTGGCTTGGAGAAGGGTTGGGGTGACACTGCGAAGCGTGTACTCGACACACTCCACTT
GCTTCTCGACCTTCTGGAGGCCCCTGATCCTGCCAACTTGGAGAAGTTCCTTGGAACTATACCAATGAT
GTTCAACGTTGTTATCCTGTCTCCTCATGGCTACTTCGCCCAGTCCAATGTGCTTGGATACCCTGACACT
GGCGGTCAGGTTGTGTACATTCTGGATCAGGTCCGTGCTTTGGAGAATGAGATGCTTCTGAGGATTAA
GCAGCAAGGCCTTGATATCACTCCGAAGATCCTCATTGTTACCAGGCTGTTGCCTGATGCTGCTGGGAC
TACGTGCGGTCAGCGGCTGGAGAAGGTCATTGGTACTGAGCACACAGACATCATTCGCGTTCCCTTCA
GAAATGAGAATGGCATCCTCCGCAAGTGGATCTCTCGTTTTGATGTCTGGCCATACCTGGAGACATACA
CTGAGGATGTTTCCAGTGAAATAATGAAAGAAATGCAGGCCAAGCCTGACCTTATCATTGGCAACTAC
AGCGATGGCAACCTAGTCGCCACTCTGCTCGCGCACAAGTTGGGAGTCACTCAGTGTACCATCGCTTA
TGCCTTGGAGAAAACCAAATACCCCAACTCGGACATATACTTGGACAAATTCGACAGCCAGTACCACT
TCTCTTGCCAGTTCACAGCTGACCTTATTGCCATGAACCACACTGATTTCATCATCACCAGCACATTCC
AAGAAATCGCGGGAAGCAAGGACACCGTGGGGCAGTACGAGTCCCACATCGCGTTCACTCTTCCTGG
GCTCTACCGTGTCGTCCATGGCATCGATGTTTTCGATCCCAAGTTCAACATTGTCTCTCCTGGAGCAGA
CATGAGTGTTTACTACCCGTATACGGAAACCGACAAGAGACTCACTGCCTTCCATCCTGAAATCGAGG
AGCTCATCTACAGCGACGTCGAGAATTCCGAGCACAAGTTCGTGCTGAAGGACAAGAAGAAGCCGAT
CATCTTCTCGATGGCGCGTCTCGACCGCGTGAAGAACATGACAGGCCTGGTCGAGATGTACGGCAAGA
ACGCGCGCCTGAGGGAGCTGGCGAACCTCGTGATCGTTGCCGGCGACCACGGCAAGGAGTCCAAGG
ACAAGGAGGAGCAGGCGGAGTTCAAGAAGATGTACAGCCTCATCGACGAGTACAAGTTGAAGGGCC
ATATCCGGTGGATCTCGGCGCAGATGAACCGCGTCCGCAACGGGGAGCTGTACCGCTACATTTGCGAT
ACGAAGGGCGCATTCGTGCAGCCTGCGTTCTACGAAGCGTTCGGCCTGACTGTGATCGAGTCCATGAC
GTGCGGTCTGCCAACGATCGCGACCTGCCATGGTGGCCCTGCTGAGATCATCGTGGACGGGGTATCTG
GCCTGCACATTGACCCTTACCACAGCGACAAGGCCGCGGATATCCTGGTCAACTTCTTTGACAAATGC
AAGGCAGATCCGAGCTACTGGGACAAGATCTCACAGGGCGGCCTGCAGAGAATTTATGAGAAGTACA
CCTGGAAGCTCTACTCCGAGAGGCTGATGACCCTGACCGGCGTGTACGGGTTCTGGAAGTACGTGAG
CAACCTGGAGAGGCGCGAGACCCGCCGCTACATCGAGATGTTCTACGCCCTGAAGTACCGTAGCCTGG
CAAGCCAGGTTCCGCTGTCCTTCGATTAG

Fig S1. The sequencing data of GRMZM2G089713 in Z58 and *sh1**. The mutation site in the *sh1** is shown in green.
