# Supplementary material for: SH1-dependent maize seed development and starch synthesis via modulating carbohydrate flow and osmotic potential balance
Source: BMC Plant Biol. 2020 Jun 8;20:264. doi: 10.1186/s12870-020-02478-1 (PMC7282075; doi:10.1186/s12870-020-02478-1)
Supplement: Supplementary file 4 — Additional file 4: Fig. S2. Full size, unedited gel used for Fig. 5c in the main text. [file 12870_2020_2478_MOESM4_ESM.pdf]

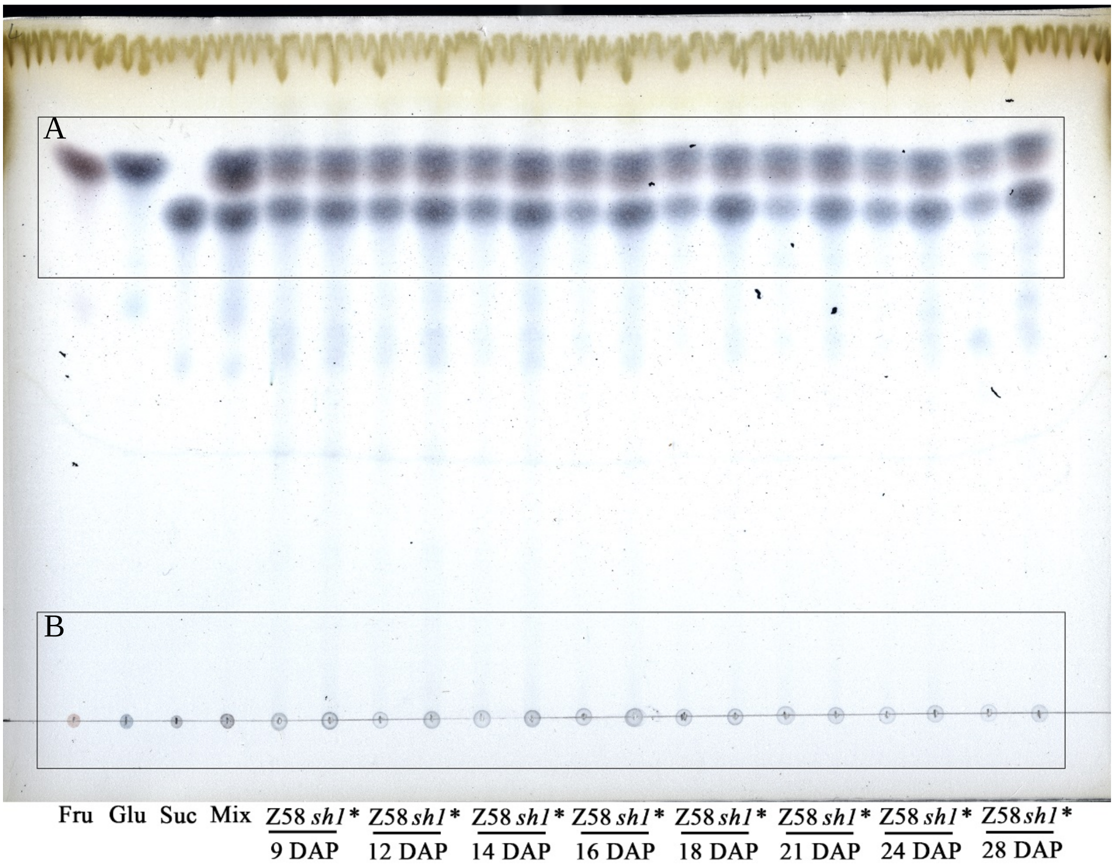


Fig S2. Full size, unedited gel used for Fig 5C in the main text. The cropped areas are labelled using black borders. A corresponds to the upper group in Fig 5C and B corresponds to the lower group in Fig 5C. Fru, Glu and Suc represented fructose, glucose and sucrose, respectively, Mix was a mixture of them.
